# Neurokinin-2 receptor antagonist SR48968 induced necroptosis of myeloid leukemia cells by calcium overload-driven reactive oxygen species accumulation

**DOI:** 10.1016/j.gendis.2023.101098

**Published:** 2023-09-20

**Authors:** Zhibin Yan, Xiangyu Hong, Qihao Lin, Leijie Wang, Gang Shao, Chentao Ge, Ruilong Xia, Caiyun Fu

**Affiliations:** a Zhejiang Provincial Key Laboratory of Silkworm Bioreactor and Biomedicine, College of Life Sciences and Medicine, Zhejiang Sci-Tech University, Hangzhou, Zhejiang 310018, China; b College of Life Sciences, China Jiliang University, Hangzhou, Zhejiang 310018, China

Increasing evidence highlight tachykinin receptors as a prominent player in hematological malignancy. We previously revealed the proto-oncogenic role of neurokinin-1 receptor (NK-1R) in acute myeloid leukemia (AML),^1^ whereas the role of neurokinin-2 receptor (NK-2R) has not been elucidated. Herein, we found NK-2R was significantly up-regulated in AML patients in The Cancer Genome Atlas databases. This result was further confirmed in blood from AML patients and a range of human leukemia cells. Then, we verified that blocking NK-2R by SR48968 markedly promoted cell death in human myeloid leukemia without cytotoxicity to normal cells. Mechanically, we uncovered that SR48968 induced cytotoxicity through necroptosis mediated by calcium overload-driven reactive oxygen species (ROS) accumulation. In summary, our results propose that NK-2R antagonist SR48968 may be used as a new therapeutic approach for myeloid leukemia.

We explored the transcription level of NK-2R in AML from The Cancer Genome Atlas datasets, and the results showed that the NK-2R expression in AML was significantly higher than that in healthy controls (Fig. S1A). Further, the expression of NK-2R was up-regulated in the AML group compared with the healthy volunteers by immunocytochemistry (Fig. 1A). The clinicopathological characteristics of the subjects are summarized in Table S1. As shown in Figure S1B, NK-2R was positively expressed in all AML patients, with strong expression in 14% of patients, moderate expression in 43% of patients, and weak expression in the remaining patients. In contrast, there was almost no NK-2R expression in healthy volunteers (Fig. 1A). Also, the expression of NK-2R was detected in NB4, HL60, and K562 cells, which the first two are human AML cell lines and the latter is chronic myeloid leukemia cell line. The results showed that NK-2R was expressed at an elevated extent in the three myeloid leukemia cell lines, while no detectable NK-2R expression was shown in four healthy volunteers (Fig. S1C). These findings suggested that NK-2R is significantly up-regulated in human myeloid leukemia. Then, we proposed the hypothesis of whether the NK-2R antagonist SR48968 can be used to inhibit the growth of leukemia cells. As expected, the methylthiazolyldiphenyl-tetrazolium bromide (MTT) assay revealed that SR48968 markedly inhibited the proliferation of K562 and HL60 in a dose- and time-dependent manner (Fig. 1B; Fig. S2A); while no significant difference was observed with respect to the human normal cluster of differentiation 34 (CD34^+^) hematopoietic cells after administrating with SR48968 (Fig. 1C; Fig. S2B, 3A). Besides, there were no obvious changes in NK-2R expression when treated with SR48968 (Fig. S3B). To gain an in-depth understanding of the role of SR48968 in the regulation of cell death, we performed flow cytometry detection assay, and the results showed that SR48968 increased the number of Annexin V-negative/PI-positive cells and Annexin V-positive/PI-positive cells, while the number of Annexin V-positive/PI-negative cells was unaltered, supporting that SR48968 may cause necroptosis in K562 and HL60 (Fig. S2C; Fig. S4A). Further, we found that propidium iodide (PI) uptake and lactate dehydrogenase leakage increased significantly after SR48968 treatment (Fig. 1D; Fig. S4B, C, 5A), which are characteristics of necrosis.^2^ Also, SR48968 induced cell cycle arrest at G_0_/G_1_ phase in K562 and HL60 (Fig. S6A). Immunoblotting showed that the enhanced phosphorylation status of receptor-interacting protein kinase 1/receptor-interacting protein kinase 3/mixed lineage kinase domain-like protein after 0.25 h of SR48968 treatment (Fig. 1E; Fig. S6B). The phenotype that SR48968 inhibited proliferation in K562 and HL60 was mostly abolished by necroptosis inhibitor necrostatin-1 (Fig. S5B, 6C). These findings strongly suggested that SR48968 induced necroptosis in human myeloid leukemia cells.

**Figure 1:**
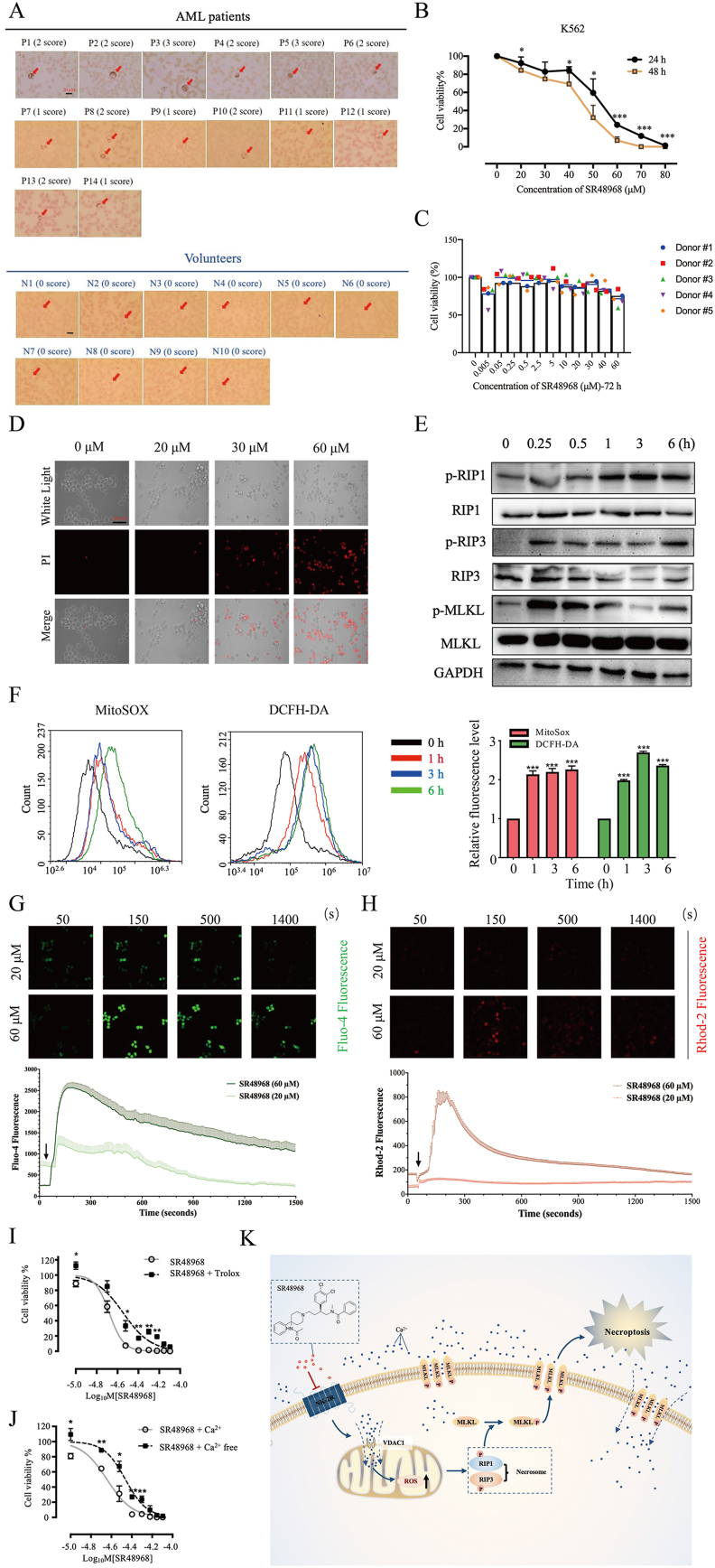
The expression level of NK-2R is up-regulated in human myeloid leukemia. **(A)** Representative immunocytochemical images of NK-2R-stained peripheral blood from AML patients and volunteers (Scale bar, 20 μm). The red arrows indicate white blood cells. **(B)** Cell viability of K562 detected by MTT assay after SR48968 treatment. ^∗^*P* < 0.05, ^∗∗^*P* < 0.01, ^∗∗∗^*P* < 0.001. **(C)** Cell viability of human normal CD34+ hematopoietic cells detected by MTT assay after SR48968 treatment for 72 h. **(D)** PI uptake by K562 cells after SR48968 treatment for 24 h (Scale bar, 100 μm). **(E)** Western blot for necroptosis-related proteins after SR48968 treatment in K562 cells. **(F)** Flow cytometry analysis of the cytoplasmic and mitochondrial ROS levels in K562 cells treatment with SR48968, and statistical relative fluorescence analysis. ^∗∗∗^*P* < 0.001. **(G)** Representative images and statistical analysis of intracellular cytosolic Ca2+ after SR48968 treatment in K562 cells by laser scanning confocal microscope. The SR48968 solution was added at the time indicated by the arrow. **(H)** Representative images and statistical analysis of intracellular mitochondrial Ca2+ after SR48968 treatment in K562 cells by laser scanning confocal microscope. The SR48968 solution was added at the time indicated by the arrow. **(I)** Effect of ROS inhibitor combined with SR48968 on K562 cell proliferation. ^∗^*P* < 0.05, ^∗∗^*P* < 0.01. **(J)** Effect of calcium inhibitor combined with SR48968 on K562 cell proliferation. ^∗^*P* < 0.05, ^∗∗^*P* < 0.01, ^∗∗∗^*P* < 0.001. **(K)** Schematic diagram of SR48968 induces cell death by inducing calcium overload/ROS-mediated necroptosis.

There are 796 significantly up-regulated proteins and 374 significantly down-regulated proteins by proteomics analysis (Table S2). The differentially expressed proteins significantly enriched in oxidative phosphorylation and ROS (Fig. S7A). The altered transcription level of ROS-related genes was shown in Figure S7B. Accumulating evidence has also indicated that ROS is immensely associated with necroptosis.^3^^,^^4^ Then, the changes of ROS in K562 were examined after administration with SR48968 by mitochondrial superoxide indicator and 2′,7′-dichlorofluorescin diacetate. As shown in Figure 1F, SR48968 promoted the accumulation of mitochondrial ROS and cytoplasm ROS in a time-dependent way in K562 cells. Besides, the inhibition effects of SR48968 in K562 and HL60 were largely abolished by trolox and mitochondrial-targeted coenzyme Q10 (Fig. 1I; Fig. S7C, D, 8), which are cytoplasm ROS and mitochondrial ROS scavengers, respectively. These findings indicated that increased ROS was an important mediator for SR48968 to exert an inhibitory effect on leukemia cells.

Ca^2+^ overload is the principal source of mitochondrial ROS increase.^5^ We detected the calcium level of intracellular calcium by using Fluo-4 AM and Rhod-2/AM. Administration of SR48968 in K562 or HL60 cells increased the calcium flow rapidly in the cytoplasm and mitochondria (Fig. 1G, H; Fig. S9A, B). To determine whether the calcium overload is necessary for SR48968 to inhibit the proliferation of leukemic cells, we pretreated K562 and HL60 cells with Ca^2+^ chelator BAPTA-AM, endoplasmic reticulum Ca^2+^-gated IP3R inhibitor 2-aminoethyl diphenylborinate (2-APB), mitochondrial Ca^2+^-blocker disodium 2,2'-(E)-ethene-1,2-diylbis(5-isothiocyanatobenzenesulfonate) (DIDS), and Ca^2+^-free medium, separately. MTT assay revealed that BAPTA-AM, DIDS, and Ca^2+^-free medium, not 2-APB, reversed the inhibitory effect of SR48968 (Fig. S9C, 10A). It indicated that Ca^2+^ in the endoplasmic reticulum is not the main reason for the increase of cytosolic calcium and mitochondrial calcium. As expected, when analyzing the ROS level by flow cytometry, the results further supported that BAPTA-AM, DIDS, and Ca^2+^-free medium, not 2-APB, reversing the effect of SR48968 on ROS production in K562 and HL60 (Fig. S9D, 10B). Taken together, these data indicated that SR48968 induced necroptosis mediated by calcium overload-driven ROS accumulation.

In conclusion, we uncover the protooncogene role of NK-2R in myeloid leukemia cells. Further, our findings suggested that NK-2R antagonist SR48968 inhibited the proliferation of myeloid leukemia cells by augmenting mitochondrial calcium overload and expediting ROS production, consequently inducing necroptosis (Fig. 1K), thereby shedding light on the potential value of targeting NK-2R as a therapeutic strategy to combat leukemia.

## Author contributions

CYF supervised the studies and provided financial support. ZBY and XYH designed the research and drafted the manuscript. QHL and LJW undertook the data analysis. GS, CTG, and RLX edited and revised the manuscript.

## Conflict of interests

The authors declare no conflict of interests.

## Funding

This work was supported by grants from the Zhejiang Provincial Natural Science Foundation of China (No. LD22H310004), the “Pioneer” R&D program of Zhejiang, China (No. 2022C03005), the National Natural Science Foundation of China (No. 81770176, 82204492), and the Special Support Plan for Zhejiang Province High-Level Talents (China) (No. 2019R52011).

## References

[1] Ge C., Huang H., Huang F. (2019). Neurokinin-1 receptor is an effective target for treating leukemia by inducing oxidative stress through mitochondrial calcium overload. Proc Natl Acad Sci U S A.

[2] Koschel J., Nishanth G., Just S. (2021). OTUB1 prevents lethal hepatocyte necroptosis through stabilization of c-IAP1 during murine liver inflammation. Cell Death Differ.

[3] Vanden Berghe T., Vanlangenakker N., Parthoens E. (2010). Necroptosis, necrosis and secondary necrosis converge on similar cellular disintegration features. Cell Death Differ.

[4] Weindel C.G., Martinez E.L., Zhao X. (2022). Mitochondrial ROS promotes susceptibility to infection via gasdermin D-mediated necroptosis. Cell.

[5] Peng T.I., Jou M.J. (2010). Oxidative stress caused by mitochondrial calcium overload. Ann N Y Acad Sci.

