# Supplementary material for: Neurokinin-2 receptor antagonist SR48968 induced necroptosis of myeloid leukemia cells by calcium overload-driven reactive oxygen species accumulation
Source: Genes Dis. 2023 Sep 20;11(5):101098. doi: 10.1016/j.gendis.2023.101098 (PMC11141248; doi:10.1016/j.gendis.2023.101098)
Supplement: Multimedia component 1 [file mmc1.docx]

**Neurokinin-2 receptor (NK-2R) antagonist SR48968 induced necroptosis of** **myeloid leukemia cells by** **calcium overload-driven reactive oxygen species (ROS) accumulation**

Zhibin Yan ^a,1^, Xiangyu Hong ^a,1^, Qihao Lin ^a^, Leijie Wang ^b^, Gang Shao ^a^, Chentao Ge ^a^, Ruilong Xia ^a^, Caiyun Fu ^a, *^

^a^ Zhejiang Provincial Key Laboratory of Silkworm Bioreactor and Biomedicine, College of Life Sciences and Medicine, Zhejiang Sci-Tech University, Hangzhou, 310018, China.

^b^ College of Life Sciences, China Jiliang University, Hangzhou, 310018, China.

^1^ These authors contributed equally to this work.

* Correspondence: fucy03@zstu.edu.cn

**Running title**: SR48968 induced necroptosis of myeloid leukemia cells

**Keywords**: Neurokinin-2 receptor; SR48968; Necroptosis; Leukemia

**Material and methods**

**Reagents**

SR48968 was obtained by WuXi AppTec (China). 2-Aminoethyl diphenylborinate (2-APB) and disodium 2,2'-(E)-ethene-1,2-diylbis(5-isothiocyanatobenzenesulfonate) (DIDS) were obtained from Sigma; Necrostatin-1 (Nec-1) and BAPTA-AM were provided by TargetMol; MitoQ was obtained from MCE; Trolox was obtained from Abcam. They were dissolved in dimethyl sulfoxide (DMSO, Yeasen) and stored at −20 °C.

**Bioinformatics analysis**

The TPM format RNAseq data of TCGA and GTEx were uniformly processed by the Toil process in UCSC XENA (htps:/xenabrowsernet/datapages/), and we extracted the corresponding TCGA data of acute myeloid leukemia and the corresponding normal tissue data in GTEx. Comparing the NK-2R expression between the two groups by R package, including “stats” “car”. The data were visualized with the ggplot2 package.

**Patients and sera collection**

Human peripheral blood (PB) samples were collected from 14 AML patients and 14 healthy volunteers, recruited at the Zhejiang Provincial Hospital of TCM. Before data collection, all of the participants written informed consent. This subjects was permitted by the Institutional Research Ethics Committee of Zhejiang Provincial Hospital of TCM. The trial protocol was compliant with the Declaration of Helsinki.

**Immunohistochemistry**

The peripheral blood was fixed by formalin. Antigens were blocked with 10% goat serum at room temperature (RT) for 30 minutes. The slides were incubated with NK-2R antibody at a ratio of 1:400 at 4℃ overnight followed by the secondary antibody at a ratio of 1:400 for 1 h at RT. subsequently, washed three times using pre-cool phosphate buffer solution (PBS), incubated with 3,3-diaminobenzidine (DAB) staining (Thermo Fisher Scientific) for detecting the NK-2R expression, counterstained with Nuclear Fast Red solution (Beyotime) subsequently. Photographs were obtained under a bright-field microscope (ZEISS), and the NK-2R expression was scored using Image J analysis software that as strong staining (positive cells, >50%), moderate staining (positive cells, >20 to 50%), weak staining (positive cells, >5 to 20%), or negative staining (positive cells, <5%).

**The isolation of** **human normal CD34^+^ hematopoietic cells**

The CD34^+^ normal hematopoietic cells from peripheral blood of 5 healthy donors were isolated and purificated as previously described.

**Cell culture and treatment**

Human myeloid leukemia cells HL60, K562 and NB4 were used in this study. HL60 were obtained from the laboratory of Professor Hong-Hu Zhu of the First Affiliated Hospital, Zhejiang University School of Medicine. K562 and NB4 were gained from the the laboratory of Professor Jingbo Zhang of Chinese Academy of Medical Sciences & Peking Union Medical College. Before these experiment, the cell lines were authenticated by short tandem repeat (STR) genotyping analysis. All cell lines were cultured in 1640 supplemented with 10% FBS (Yeasen) and 1% antibiotics at 37 °C in a cell incubator 5% CO_2_ atmosphere.

The cells were cultured in 75 cm^2^ cell culture dish. For experimental purposes, cells were cultured in 6-well plates (2 × 10^5^ cells/well) or 96-well plates (1 × 10^4^ cells/well). Cells were allowed to grow for 1 h before treatment. Where specified, the chemical inhibitor was added to the media 1 h prior to SR48968 addition.

**MTT assay**

(3-(4,5-dimethylthiazol-2-yl)-2,5-diphenyltetrazolium bromide (MTT) assay was performed to evaluate the cell viability. After treatment of cells for a specific time, put in 20 μl MTT solution (5 mg/ml) under dark conditions and placed in a cell incubator for 4 h. Following centrifugation, discarded the supernatant fluid and then added 200 µl DMSO, moderate shaking for 10 min to achieve complete solubilize the formazan crystals. The viable cell numbers were quantified by the microplate spectrophotometer system (Thermo, Varioskan flash) at 570 nm.

**Western blot**

Myeloid leukemia cells after SR48968 treatment were collected and washed using pre-cool PBS, then added RIPA (Servicebio) lysate and PMSF mixture (RIPA: PMSF=100:1) in ice for 30 min, centrifuged at 4 ℃ 10000 rpm for 20 min to extract the supernatant, detected the protein concentrations using the BCA Protein Assay kit (Solarbio). The equivalent quantities of protein were denatured at 100 ℃, 5 min, then separated by SDS-PAGE gel electrophoresis, and transferred to the polyvinylidene difluoride (PVDF) membrane (Millipore). Subsequently, blocked the membrane with 5% skimmed milk for 2 h, overnight incubation of the corresponding primary antibody in a cold refrigerator. Primary antibody used were diluted as follows: NK-2R polyclonal antibody (1:1000, Biogot)；RIP1 recombinant rabbit monoclonal antibody (1:1000, HuaBio)；RIP3 antibody produced in rabbit (1:1000, Sigma)；MLKL recombinant rabbit monoclonal antibody (1:1000, HuaBio); Phospho-RIP rabbit monoclonal antibody (p-RIP1, 1:1000, CST); Phospho-RIP3 rabbit monoclonal antibody (p-RIP3, 1:1000, CST); Phospho-MLKL rabbit monoclonal antibody (p-MLKL, 1:1000, CST); GAPDH polyclonal antibody (1:1000, Biogot); β-actin polyclonal antibody (β-actin, 1:1000, Biogot). Incubation with appropriate peroxidase-conjugated secondary antibodies (1:10000, Biogot) for 2 h at RT. The antigen-antibody complexes were visualized through an electrochemiluminescence detection system (ChampChemi 910 Plus, Sagecreation). Quantification of the band intensities was performed using ImageJ software (Bio-Rad, USA).

**Calcium imaging**

Fluo-4 AM (Yeasen) and Rhod-2 AM (Yeasen) were used to measure the dynamic changes in cytosolic and mitochondrial Ca^2+^ concentrations respectively. Before staining as manufacturer’s instructions, cells were coated with 0.01% poly-L-lysine at RT for 30 min and washed three times using Ca^2+^ recording buffer. Calcium was imaged by Laser Scanning Confocal Microscope (CISI, Nikon). Fluorescence was screened at 5 seconds intervals throughout stimulation.

**LDH assay**

Changes in LDH release were detected by LDH Release Assay Kit (Beyotime) with multimode reader (Varioskan Flash) according to the manufacturer’s instructions.

**PI uptake capacity**

Propidium iodide (PI) (Solarbio) was used for the measurement of plasma membrane permeability. After SR48968 treatment, then cells added 1.5 μM PI dilution and incubated in the instrument at 37°C with 5% CO_2_ for 30 min. The change of red fluorescence in the cells was observed by Inversed Fluorescent Microscope (Axio Vert. A1, ZEISS).

**Proteomics analysis**

Labeling and preparation of the samples by stable isotope labeling with amino acids in cell culture (SILAC) as previously described. ^1^ Briefly, the K562 cells were labeled with either “heavy isotopic lysine” (^13^C-Lysine) or “light isotopic lysine” (^12^C-Lysine) using a SILAC Protein Quantitation Kit (Pierce, Thermo) according to manufacturer’s instructions. Proteins were extracted and digested with trypsin, HPLC fractionation followed by high-resolution LC-MS/MS analysis, quantitative global proteome analysis was performed. We set quantification ratio of >1.3 as up-regulated threshold and <0.77 as down-regulated threshold.

**Measurement of ROS**

The 5 μM peroxide-sensitive fluorescent probe DCFH-DA (Beyotime) and 5 μM mitochondrial superoxide indicator MitoSOX (Thermo) were used to detect intracellular cytosolic and mitochondrial ROS respectively with a flow cytometer.

**Flow cytometry (FCM)**

After treatment with SR48968 for 24 hours, cells were harvested, centrifuged, and resuspended in PBS. The apoptosis assay was performed by the Annexin V-FITC Apoptosis Detectio (Keygen), according to the manufacturer's protocol. For cell cycle assay, cells were stored in 70% ethanol overnight at -20 °C, then stained with Cell cycle staining buffer (RNase A: PI=1:9) in a dark environment for 30 min. Then, analyzed the cell cycle distribution or cell apoptosis rate by the Guava® easyCyte Flow Cytometer (BD, Accuri C6). A total of 10000 events were acquired for each sample.

**Statistical analysis**

Individual experiment was carried out at least three times. All data were presented in the form of mean ± standard error of the mean (SEM) and analyzed by GraphPad Prism 9.0 (San Diego). Student’s t-tests was applied to compare the differences between the two groups, while the differences among more than two groups were performed through one-way analysis of variance (ANOVA) followed by Tukey’s test. In all analysis, * *P* < 0.05, ** *P* < 0.01, and *** *P* < 0.001 were considered statistically significant.

**Figures**

**Figure S1**

**
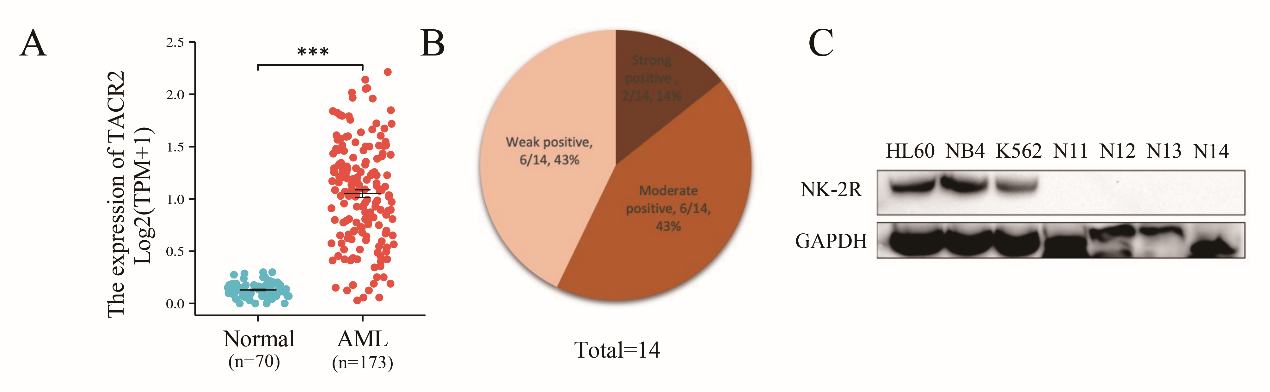
**

**Figure S1. The expression level of NK-2R is upregulated in human myeloid leukemia. (A)** The expression level of TACR2 in normal and AML samples in TCGA. ****P<*0.001. **(B)** Pie chart overview of NK-2R positivity in immunostained AML samples (n = 14). **(C)** Western blot of NK-2R expression in HL60, NB4, K562 and healthy peripheral blood cells.

**Figure S2**

**
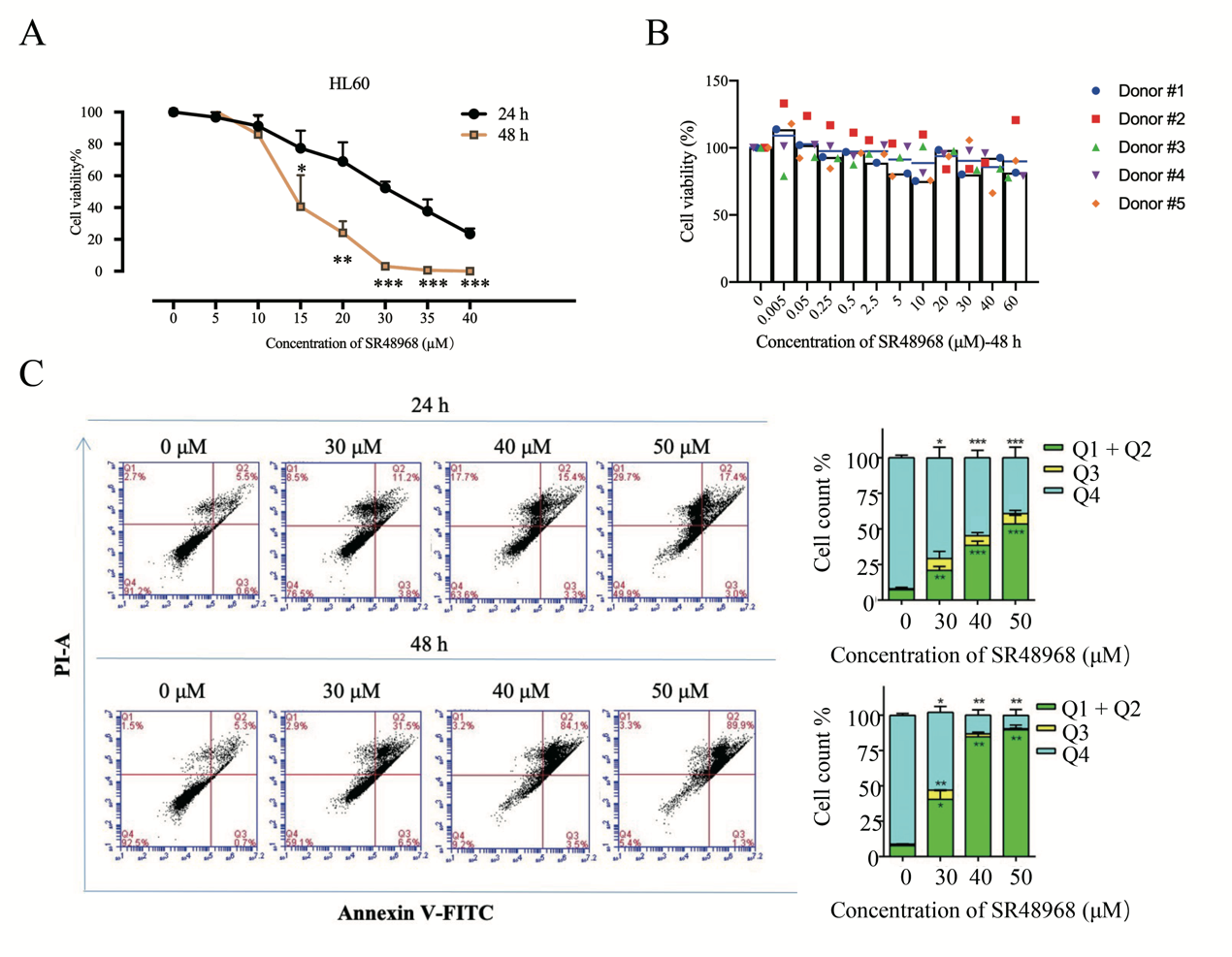
**

**Figure S2. NK-2R antagonist SR48968 triggers cell death in myeloid leukemia cells. (A)** Cell viability of HL60 cells detected by MTT assay after treatment of SR48968. **P<*0.05, ***P<*0.01, ****P<*0.001. **(B)** Cell viability of human normal CD34^+^ hematopoietic cells detected by MTT assay after SR48968 treatment. **(C)** The distribution of FITC and PI fluorescence in K562 cells in flow cytometry after SR48968 treatment for 24 h and 48 h, and its statistical analysis. **P<*0.05, ***P<*0.01, ****P<*0.001.

**Figure S3**

**
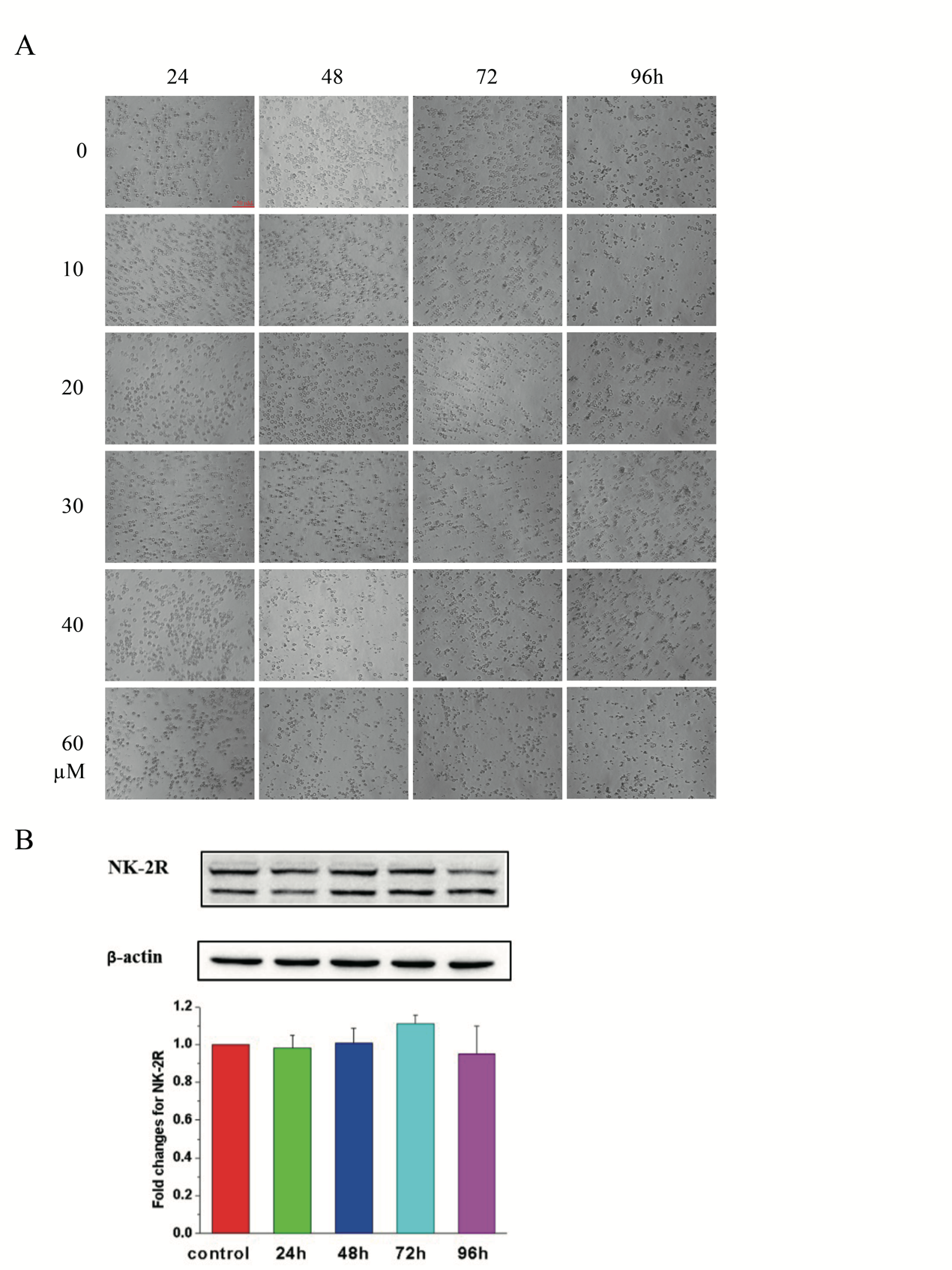
**

**Figure S3. The effect of SR48968 on the human normal CD34^+^ hematopoietic cells and the expression of NK-2R.**

**(A)** Typical optical micrographs of human normal CD34^+^ hematopoietic cells treated by SR48968 at different concentrations and time points (Scale bar, 50 μm). **(B)** Representative pictures of Western blot (upper) and columnar statistics analysis (lower) of K562 cells treated by SR48968 at 24 h, 48 h, 72 h and 96 h, as well as the control.

**Figure S4**

*
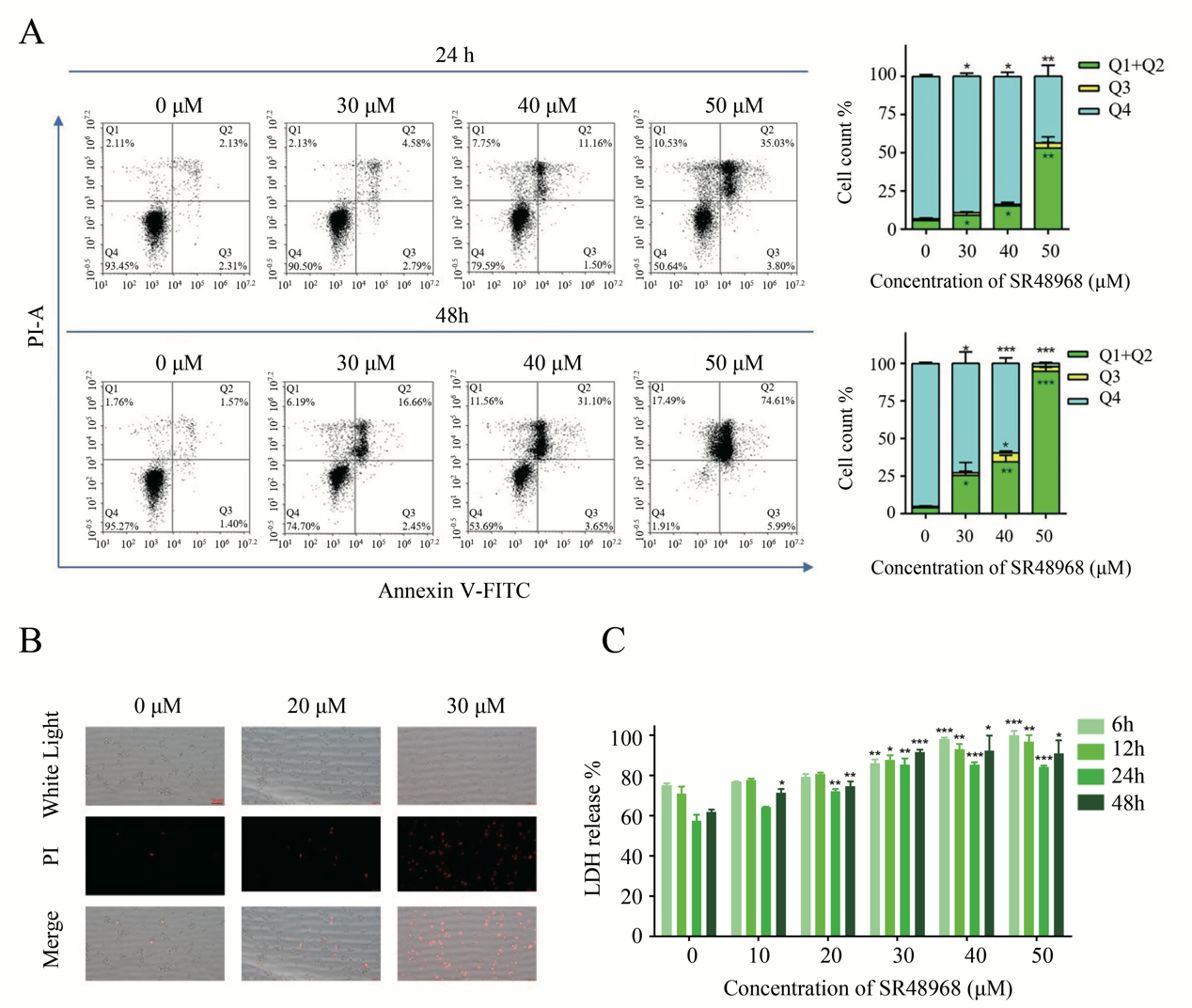
*

**Figure S4. NK-2R antagonist SR48968 triggers cell death in HL60 cells.**

**(A)** The distribution of FITC and PI fluorescence in HL60 cells by flow cytometry after treatment with different concentrations of SR48968 for 24 h and 48 h, and its statistical analysis. **P*<0.05, ***P*<0.01, ****P*<0.001. **(B)** PI uptake by HL60 cells after treatment with different concentrations of SR48968 for 24 h (Scale bar, 50 μm). **(C)** Changes in LDH release from H60 cells after treatment with different concentrations of SR48968 at various time points. **P*<0.05, ***P*<0.01, ****P*<0.001.

**Figure S5**

**
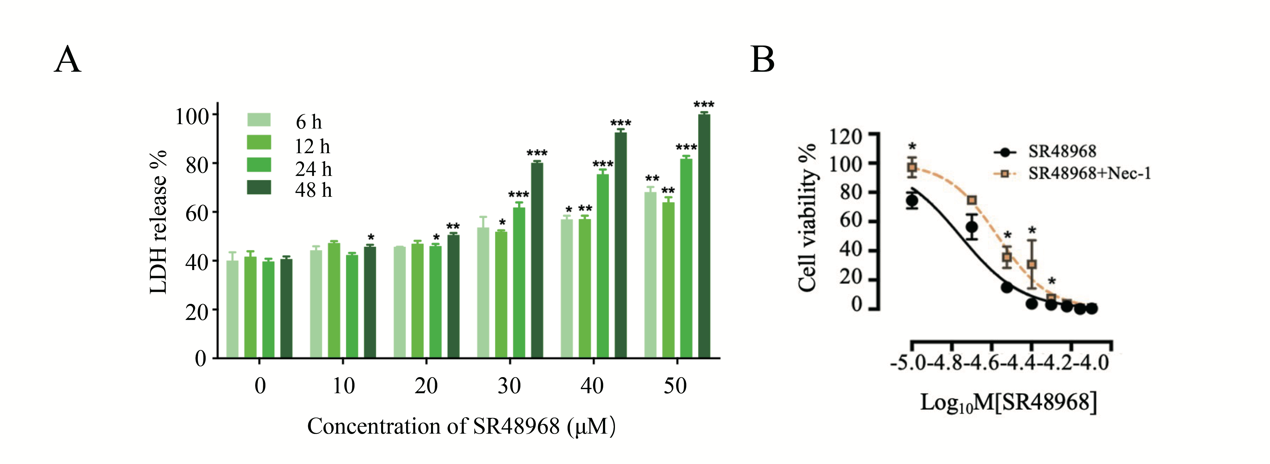
**

**Figure S5. SR48968 kills myeloid leukemia cells by necroptosis in K562 cells.** **(A)** Changes in LDH release from K562 cells after treatment with SR48968. **P<*0.05, ***P<*0.01, ****P<*0.001. **(C)** Cell viability of necrosis inhibitor Nec-1 on the proliferation of K562 cells induced by SR48968. **P<*0.05.

**Figure S6**

***
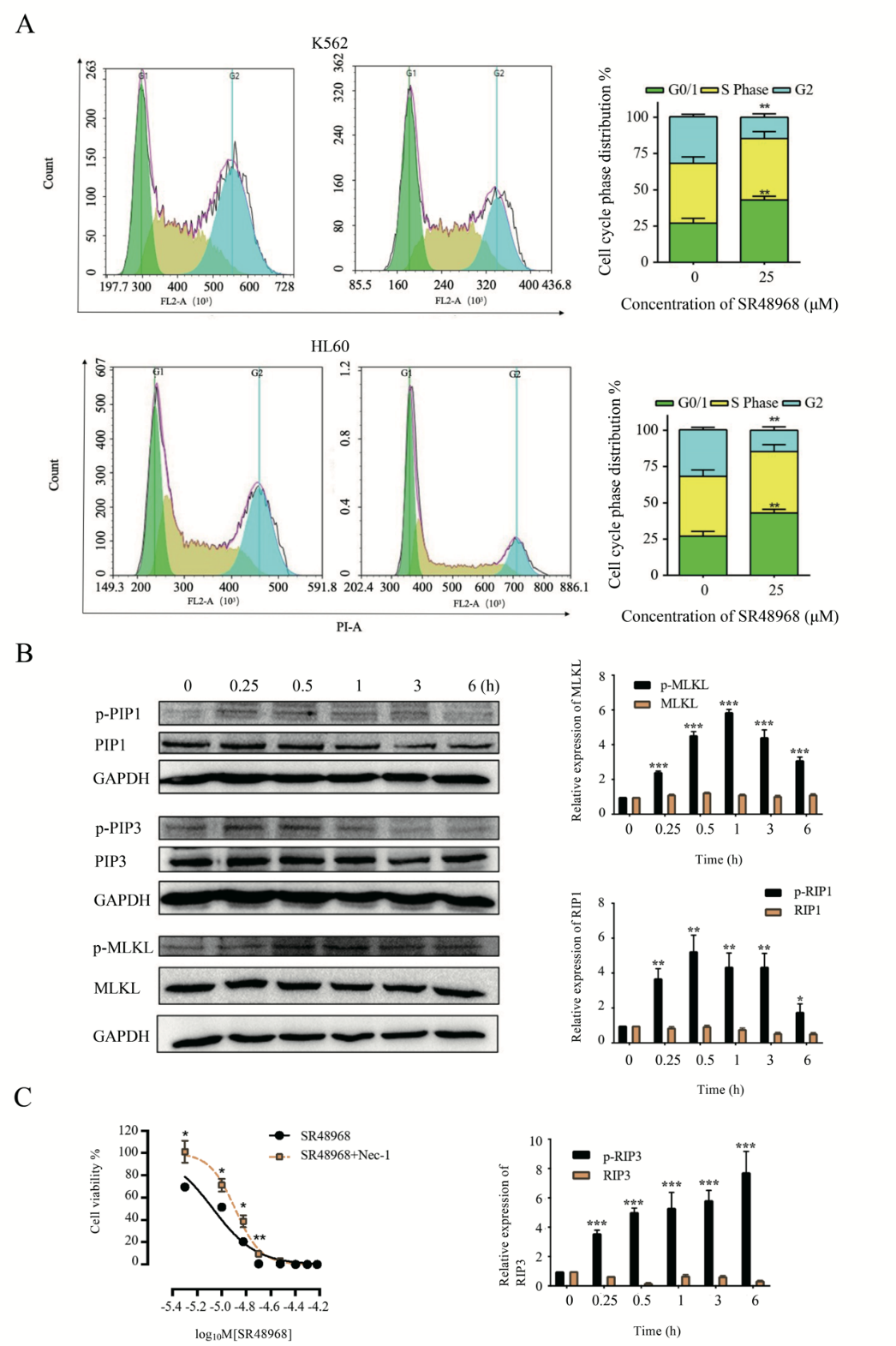
***

**Figure S6. SR48968 kills myeloid leukemia cells by necroptosis.**

**(A)** The cyclic change representative map of K562 and HL60 cells, and statistical chart of K562 and HL60 cell cycle phase distribution after SR48968 treatment (25 μM). ***P*<0.01. **(B)** Western blot for necrotic-related proteins after treatment of HL60 cells with SR48968, and its statistical analysis. **P*<0.05, ***P*<0.01, ****P*<0.001. **(C)** Cell viability of necrosis inhibitor Nec-1 on the proliferation of HL60 cells induced by SR48968. **P*<0.05, ***P*<0.01.

**Figure S7**


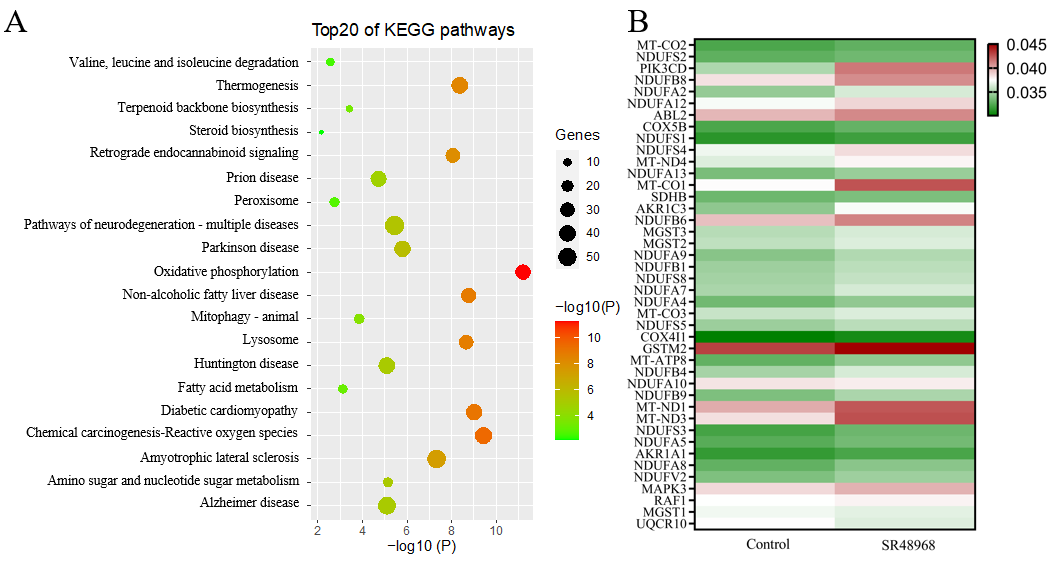


**Figure S7.** **Proteomics analysis after 24 h treatment of SR48968 in K562 cells.** **(A)** Top 20 enriched KEGG pathways of the DEPs. **(B)** Heatmap showed the ROS-related proteins expression.

**Figure S8**

*
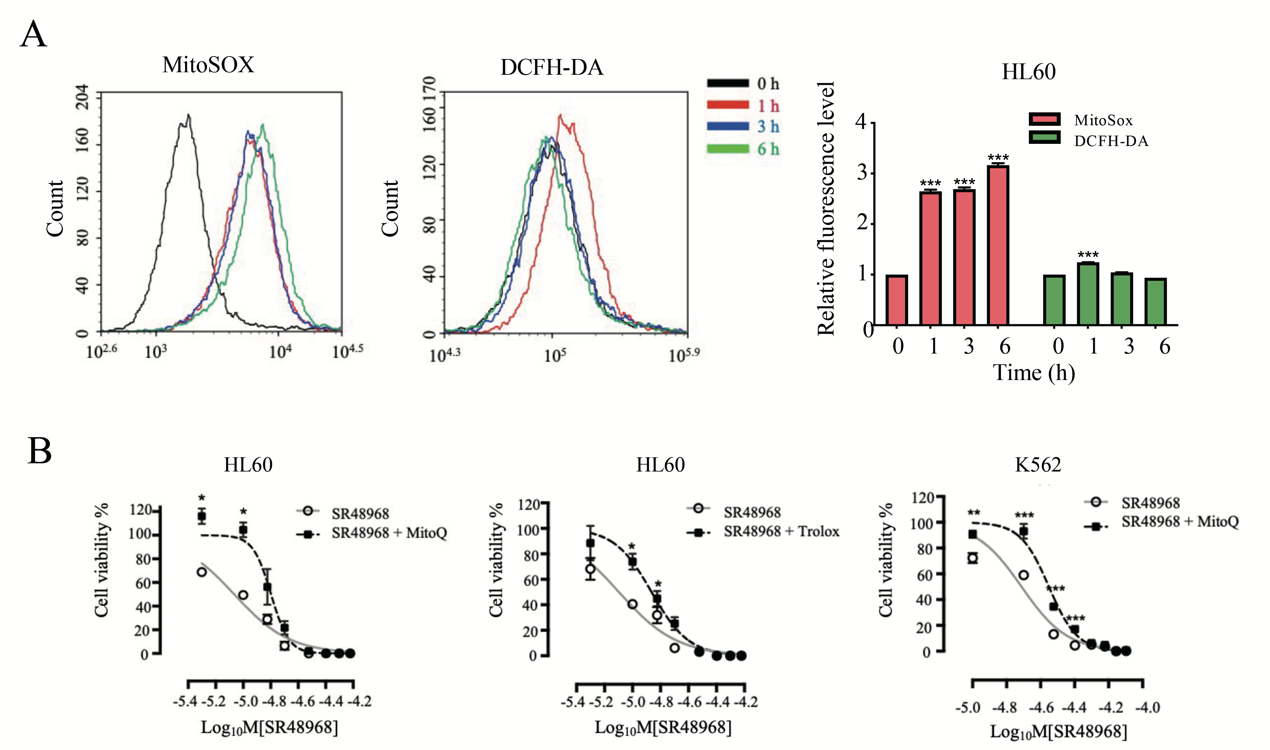
*

**Figure S8. SR48968 promotes ROS accumulation in human myeloid leukemia cells.**

**(A)** Representative images of flow cytometry analysis of the cytoplasmic and mitochondrial ROS levels in K562 cells treated with SR48968, and statistical relative fluorescence analysis. ****P*<0.001. **(B)** Effect of pretreatment with reactive oxygen scavenger on the viability of HL60 and K562 cells after SR48968 treatment. **P*<0.05, ***P*<0.01, ****P*<0.001.

**Figure S9**


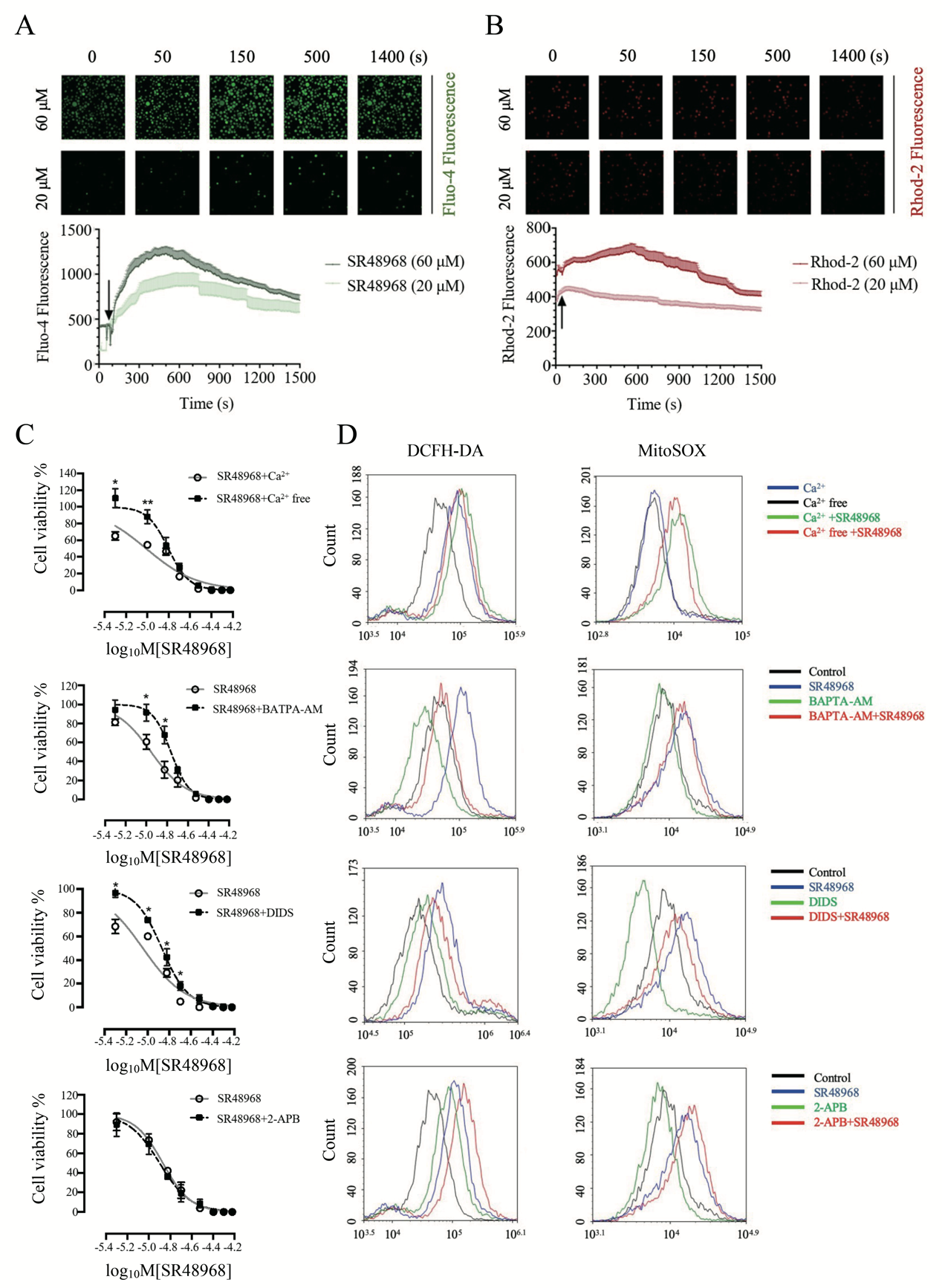


**Figure S9. Cytoplasmic and mitochondrial Ca^2+^ influx induced by SR48968 increased cytoplasmic and mitochondrial ROS level in HL60 cells.**

1. Representative images and statistical analysis of intracellular cytosolic Ca^2+^ after treatment with SR48968 in HL60 cells by laser scanning confocal microscope. The SR48968 solution was added at the time indicated by the arrow. **(B)** Representative images and statistical analysis of intracellular mitochondrial Ca^2+^ after SR48968 treatment in HL60 cells by laser scanning confocal microscope. The SR48968 solution was added at the time indicated by the arrow. **(C)** Effect of calcium inhibitor combined with SR48968 on HL60 cell proliferation. **P*<0.05, ***P*<0.01. **(D)** Representative images of flow cytometry analysis of the cytoplasmic and mitochondrial ROS levels in K562 cells treated with SR48968 in the absence of calcium and calcium, and after pretreatment with Ca^2+^ chelators BAPTA-AM (10 μM), DIDS (20 μM) and 2-APB (10 μM).

**Figure S10**

**
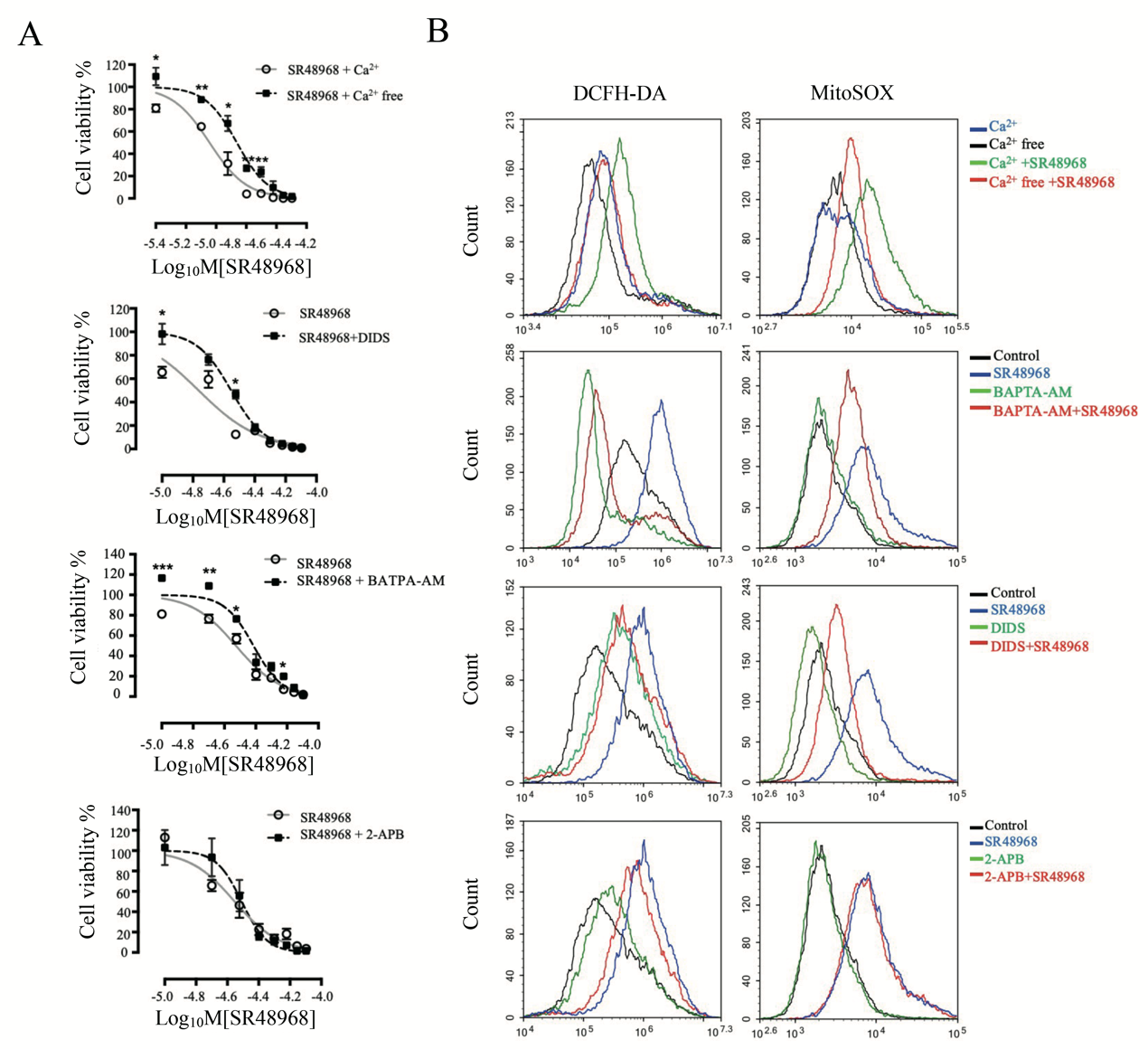
**

**Figure S10. Cytoplasmic and mitochondrial Ca^2+^ influx induced by SR48968 increased ROS level in K562 cells.** **(A)** Effect of calcium inhibitor combined with SR48968 on K562 cell proliferation. **P<*0.05, ***P<*0.01, ****P<*0.001. **(B)** Representative images of flow cytometry analysis of the cytoplasmic and mitochondrial ROS levels in K562 cells treated with SR48968 in the absence of calcium and calcium, and after pretreatment with Ca^2+^ chelators BAPTA-AM (10 μM), DIDS (20 μM) and 2-APB (10 μM).

**Table S1**. This study included 14 cases of de novo AML (M2, n = 5; M3, n = 1; M4, n = 1; M5, n = 7, according to the French-American-British classification).

| Patient | Age | Gender | Stage | WBC (×10^9^/L) | NK-2R score |
| --- | --- | --- | --- | --- | --- |
| 1 | 75 | female | M5 | 7.4 | 2 |
| 2 | 27 | male | M3 | 10.0 | 2 |
| 3 | 47 | male | M5 | 225.0 | 3 |
| 4 | 69 | female | M2a | 4.1 | 2 |
| 5 | 45 | male | M5 | 2.1 | 3 |
| 6 | 69 | male | M5 | 5.4 | 2 |
| 7 | 27 | male | M4E0 | 2.57 | 1 |
| 8 | 60 | female | M2 | 50.0 | 1 |
| 9 | 75 | male | M2 | 1.3 | 1 |
| 10 | 27 | male | M5b | 33.8 | 2 |
| 11 | 43 | female | M5 | 2.4 | 1 |
| 12 | 29 | female | M2a | 15.65 | 1 |
| 13 | 44 | female | M5 | 12.15 | 2 |
| 14 | 79 | male | M2 | 17.02 | 1 |

WBC, white blood cell.
